# Development of fatty liver disease model using high cholesterol and low choline diet in white leghorn chickens

**DOI:** 10.1007/s11259-024-10420-1

**Published:** 2024-06-11

**Authors:** Kush Kumar Yadav, Patricia A. Boley, Saroj Khatiwada, Carolyn M. Lee, Menuka Bhandari, Scott P. Kenney

**Affiliations:** 1 Center for Food Animal Health (CFAH), Department of Animal Sciences, The Ohio State University, 1680 Madison Ave, Wooster, OH 44691 USA; 2 Department of Veterinary Preventive Medicine, The Ohio State University, Columbus, OH 43210 USA

**Keywords:** Chicken, Fatty liver, Model, Diet-induced

## Abstract

Nonalcoholic fatty liver disease (NAFLD), which shows similar symptoms as fatty liver hemorrhage syndrome (FLHS) in chickens, is the most common cause of chronic liver disease and cancer in humans. NAFLD patients and FLHS in chickens have demonstrated severe liver disorders when infected by emerging strains of human hepatitis E virus (HEV) and avian HEV, respectively. We sought to develop a fatty liver disease chicken model by altering the diet of 3-week-old white leghorn chickens. The high cholesterol, and low choline (HCLC) diet included 7.6% fat with additional 2% cholesterol and 800 mg/kg choline in comparison to 5.3% fat, and 1,300 mg/kg choline in the regular diet. Our diet induced fatty liver avian model successfully recapitulates the clinical features seen during NAFLD in humans and FLHS in chickens, including hyperlipidemia and hepatic steatosis, as indicated by significantly higher serum triglycerides, serum cholesterol, liver triglycerides, cholesterol, and fatty acids. By developing this chicken model, we expect to provide a platform to explore the role of lipids in the liver pathology linked with viral infections and contribute to the development of prophylactic interventions.

**Supplementary Information:**

The online version contains supplementary material available at 10.1007/s11259-024-10420-1.

## Introduction

Non-alcoholic fatty liver disease (NAFLD) causes chronic liver disease in humans which is an emerging major human health concern of the modern world (Chalasani et al. 2018; Younossi et al. 2020). NAFLD is characterized by hepatic steatosis (fat buildup) in the absence of any defined reasons for fat deposition (e.g., excessive alcohol consumption, viral hepatitis, drug-induced liver injury). Histologically, NAFLD is the accumulation of 5 to 10% fat in the liver (Caldwell et al. 2009). One or more components of metabolic syndromes like systemic hypertension, dyslipidemia, insulin resistance, fat accumulation, mitochondrial dysfunction (Zhang et al. 2023), oxidative stress, ER stress and ROS production (Dai et al. 2022; Jiang et al. 2024) in association with genetic or epigenetic factors can be a predisposing risk factor to NAFLD (Flessa et al. 2022). NAFLD is the second leading reason for liver transplantation in the United States (US), behind only alcohol-related liver disease (Kim et al. 2018). About 25% of people have NAFLD globally, among which 80 million cases have been reported only in the US. (Younossi et al. 2016). By 2030, NAFLD is estimated to be present in 100 million people in the US, based on obesity percentage, type 2 diabetes mellitus (T2DM), and the increasing elderly population (Estes et al. 2018). In addition, cardiovascular diseases are related to visceral obesity, which is also a factor in NAFLD (Cotter and Rinella 2020). Thus, the rise in the NAFLD necessitates a rapid fatty liver disease animal model that can be used to study NAFLD and in comorbidity studies. Currently, existing animal models for NAFLD replicate either the histopathology or physiological properties of NAFLD (Soret et al. 2020). Hence, it is crucial to develop an animal model that can rapidly demonstrate both the physiological and histopathological properties similar to NAFLD in humans.

Fatty liver hemorrhage syndrome (FLHS) frequently occurs in caged high-production laying hens (peaking at 33 weeks of age) and is an economically important disease in the poultry industry worldwide. It is also a leading cause of death by noninfectious agents in backyard chickens (Trott et al. 2014). In general, laying hens with FLHS have a drop in egg production and sudden mortality (Trott et al. 2014). FLHS in chickens is characterized by hyperlipidemia similar to dyslipidemia seen in steatohepatitis in humans (Du et al. 2016; Gao et al. 2019). Furthermore, FLHS in laying hens produces an increase in plasma dipeptidyl peptidase 4 (DPP4), an enzyme associated with NAFLD in humans (Baumeier et al. 2017; Tsai et al. 2017). In addition, the aged laying hen model has been used to understand the underlying metabolic mechanism of taurine alleviating NAFLD, suggesting taurine’s application in the prevention of NAFLD in humans (Yu et al. 2023). Thus, chickens could be a useful animal model to study NAFLD comingled with viral infections.

Common animal models for HEV research include primates, chickens, pigs, rabbits, and more recently mice and gerbils (Kenney and Meng 2019; Li and Wakita 2019; Yadav and Kenney 2023). The liver is the only site for de novo lipogenesis in both chickens and humans, whereas in lagomorphs and rodents, both adipose tissue and liver contribute to de novo lipogenesis (Laliotis et al. 2010; Letexier et al. 2003). Interestingly, in pigs, which are the most utilized biomedical model for HEV infection, the primary de novo lipogenesis site is the adipose tissue (Bergen and Mersmann 2005). Models of NAFLD in chickens would be a novel approach for studying viral diseases that cause chronic hepatitis in conjunction with fatty liver disease. Acute on chronic hepatitis has been reported in humans infected with HEV that have the underlying NAFLD (Davern et al. 2011; Lenggenhager et al. 2021). However, the mechanism by which HEV aggravates the NAFLD causing lethal scenario in humans has not been elucidated. Thus, this study is the initial step to develop a diet induced chicken model which will be further utilized to determine the role of HEV in severe liver disease pathogenesis.

Currently, all available chicken fatty liver disease models are time consuming (Ayala et al. 2009; Rozenboim et al. 2016; Zhang et al. 2011). A fast and dependable avian fatty liver disease model is needed to develop therapeutic drugs and to understand differing comorbidities related to viral hepatitis. Here, we demonstrate a chicken model of fatty liver disease that occurs within a 5-week period by altering their diet with high cholesterol and low choline.

Cholesterol is an essential building block for human tissues (Craig et al. 2023). Steatohepatitis and hepatic inflammation in animal models (Matsuzawa et al. 2007; Subramanian et al. 2011; Zheng et al. 2008) and humans (Savard et al. 2013) has been linked to dietary cholesterol. In addition, an important nutrient, choline, is stored and metabolized in the liver. Hepatic very‐low‐density lipoprotein (VLDL) secretion is hampered by reducing choline in animal diets and results in hepatic steatosis, oxidative stress, liver cell death, and changes in cytokines and adipocytokines (Corbin and Zeisel 2012). Thus, we hypothesized that diet-induced fatty liver disease can be developed in chickens to understand the pathophysiology of NAFLD and viral disease comorbidities in order to develop prophylactic targeted interventions against them.

## Materials and methods

### Experimental design

Animal experiments in this study were approved by The Ohio State University Institutional Animal Care and Use Committee. A total of 16 specific pathogen free (SPF) white leghorn chickens irrespective of sex were divided randomly into two groups of 8 each. The birds were floor housed with wood shavings as litter and provided ad libitum access to feed and water. The birds were raised in appropriate environmental conditions: 83–85 °F room temperature (age—day 21 to 27), followed by 78–80 °F (day 28 to 34), 73–75 °F (day 35 to 41), 68–70 °F (day 42 to 48) and 65–68 (day 49 to till the end of the experiment). Feed formulation (Lin et al. 2021) is provided in the supplementary data (Tables S1 and S2).

Standard floor housing management practices were used throughout the experimental period. Birds and facilities were inspected twice daily. The following data were recorded: general health status of chickens, room temperature, and constant feed and water supply. Half of the birds in each group (*n* = 4) were humanely euthanized on day 17 and the remaining birds were euthanized on day 35. After euthanasia, the body weight of each bird was measured prior to necropsy. Liver specific gross lesions were recorded on day 17 and day 35 during the necropsy.

### High cholesterol and low choline (HCLC) diet

Major components of the control/regular diet include 17% protein, 5.3% fat, and 1300 mg/kg choline. In contrast, the HCLC diet includes 17% protein, 7.6% fat with an additional 2% cholesterol and 800 mg/kg choline (Lin et al. 2021) (Table 1). Detailed information on the feed formulation is available in Supplementary data (Table S1). In general, the recommended dosage for fat and choline is dependent upon the breed and age of the chicken. In general, for day 22 to 56 day-old chickens, the range of choline is from 1083.45 mg/kg to 1241.85 mg/kg (Ramalho de Lima et al. 2024). In addition, the recommended fats in poultry nutrition are from 20 to 50 g/kg (5%) (Ravindran et al. 2016).

**Table 1:** Major difference in the dietary composition in chicken feed

Group 1 (Regular Diet)		Group 2 (High Cholesterol and Low choline diet)	
Protein	17%	Protein	17%
Fat	5.3%	Fat	7.6% with additional 2% cholesterol
Choline	1300 mg/kg	Choline	800 mg/kg
*n* = 8		*n* = 8	

#### Liver organ index (LOI)

The liver from each bird was weighed and recorded. Briefly, the liver was surgically separated from other organs during necropsy of euthanized animals. Careful attention was given that additional tissue was not attached to the liver. LOI was calculated using the following formula:

$$\text{Liver organ index}\left(\text{LOI}\%\right)=\left[\frac{\text{Liver weight of a chicken}}{\text{total body weight of a chicken}}\right]\times{100\%}$$

### Serum triglycerides and cholesterol evaluation

Chicken blood samples were collected from brachial wing veins. The blood samples were centrifuged at 3000 revolutions per minute (rpm) for 10 min in a Sorvall Legend XTR Centrifuge (relative centrifugal force of 1.08 × g) at room temperature to collect the separated serum. The serum fraction was subjected to triglycerides colorimetric (Cayman chemical) and cholesterol fluorometric (Cayman chemical) measurements. Absorbance was measured at 535 nm for triglycerides. Fluorescence was read using excitation wavelength of 535 nm and emission wavelength of 590 nm for cholesterol. Detection kits were used according to the manufacturer’s instructions. The signals were detected using a microplate spectrophotometer (Molecular Devices).

### Liver triglycerides, cholesterol, and fatty acid measurement

Liver samples were collected from each group post weighing and stored using a snap freeze technique in liquid nitrogen. Twenty milligrams of frozen chicken liver were homogenized in 1 ml of phosphate buffered saline (PBS). Five milliliters of chloroform/methanol (2/1, volume/volume) were added to each sample. Samples were vortexed for 1 min followed by incubation at 4 °C for 2 h. The bottom phase was collected after centrifugation at 1,650 × g for 10 min and was allowed to dry. Isopropanol/NP-40 solution (9:1) was used to dissolve the lipid samples before the assay. Liver triglycerides, cholesterol, and fatty acid concentrations were measured using triglycerides colorimetric- (Cayman chemical), cholesterol fluorometric- (Cayman chemical), and fatty acid fluorometric- (abcam) measurement kits. For cholesterol and triglycerides, a similar wavelength as described above was used. For fatty acids measurements, fluorescence was read using excitation wavelength of 535 nm and emission wavelength of 590 nm. The signals were detected using a microplate spectrophotometer (Molecular Devices). Data were normalized with the tissue weight.

### Histological analysis

Liver samples were fixed in 10% neutral buffered formalin. Liver sections were paraffin-embedded and sliced at 4 µm thick sections and stained with hematoxylin and eosin. The tissue sections were examined and compared with negative controls.

### Statistical analysis

Quantitative data are presented with the mean and standard deviation. The student’s unpaired two-tailed t test was used to analyze the data. Statistically significant differences (*P* < 0.05) between the groups were computed using GraphPad Prism 9.4.1.

## Results

### HCLC induced hyperlipidemia in specific pathogen free white leghorn chickens

**Fig. 1:**
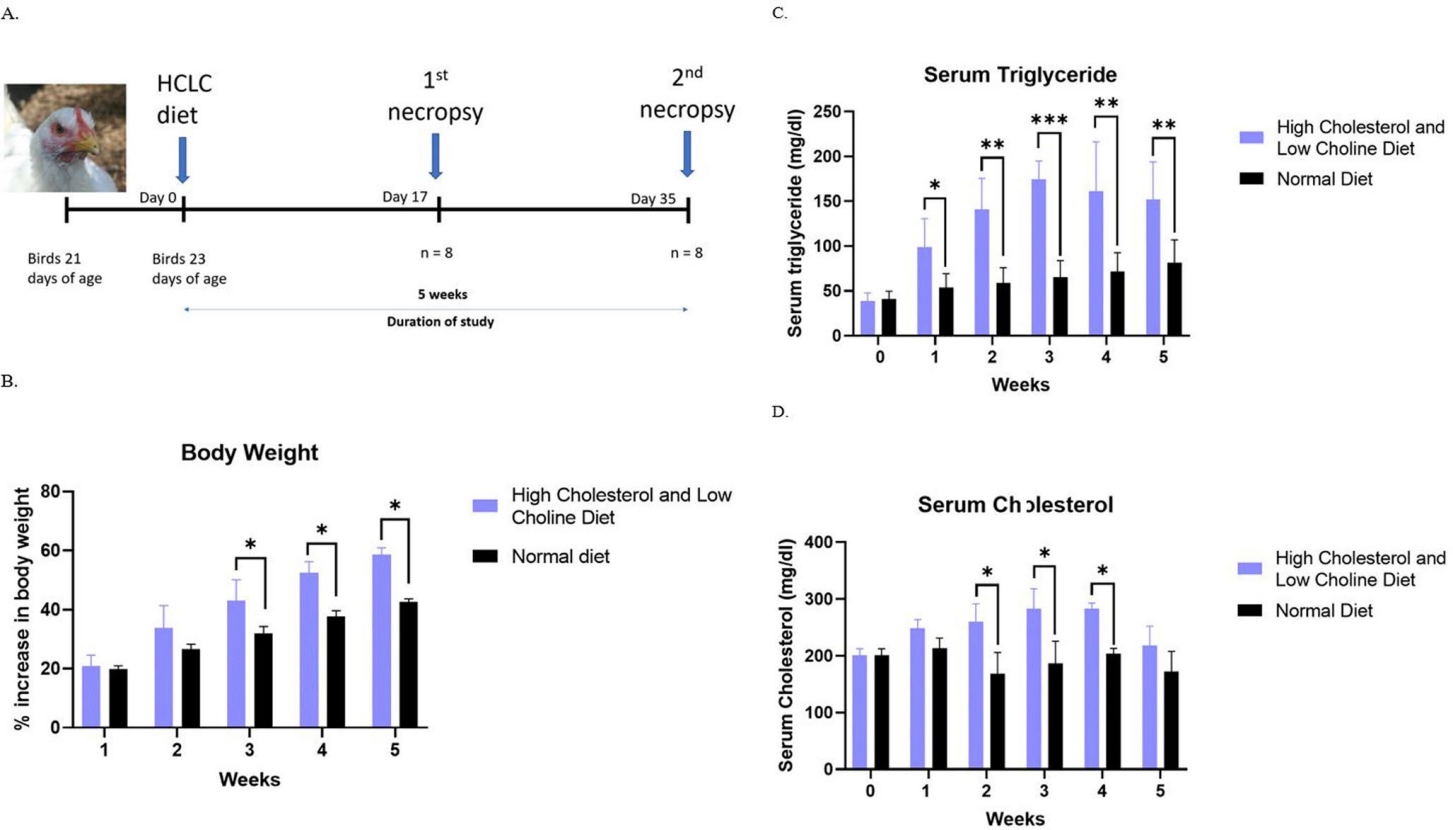
HCLC diet induced hyperlipidemia in white leghorn breed chickens. **A** Schematic diagram demonstrating the experimental design. A total of 16 chickens were used in the study, eight in each group. **B** Percentage of increase in body weight was observed in HCLC fed chickens showing significant differences starting at week 3 until five weeks. **C** Serum triglycerides were increased in HCLC fed chickens starting week 1. **D** Serum cholesterol was increased in HCLC fed chickens showing significant differences starting at week 2 until week 4. Data are shown as mean ± SD. HCLC, high cholesterol and low choline diet. * *P* < 0.05, ** *P* < 0.01, *** *P* < 0.001

At 23 days of age, birds were subjected to the HCLC diet for 5 weeks (Fig. 1A). Differences in body weight were observed between the groups starting from the third week to the fifth week of the study. (Fig. 1B). After one week, a statistically significant difference in the levels of triglycerides was detected between groups. The difference was consistent until the end of the study on the fifth week (Fig. 1C). The difference in the cholesterol concentration between the two groups was similar to the triglycerides with elevated levels observed from week 1 through week 5, however, only the second, third, and fourth weeks of the study had significant differences (Fig. 1D). These findings demonstrate hyperlipidemia in the white leghorn chicken when fed with HCLC diet.

### HCLC diets induced hepatic steatosis in SPF white leghorn chickens

**Fig. 2:**
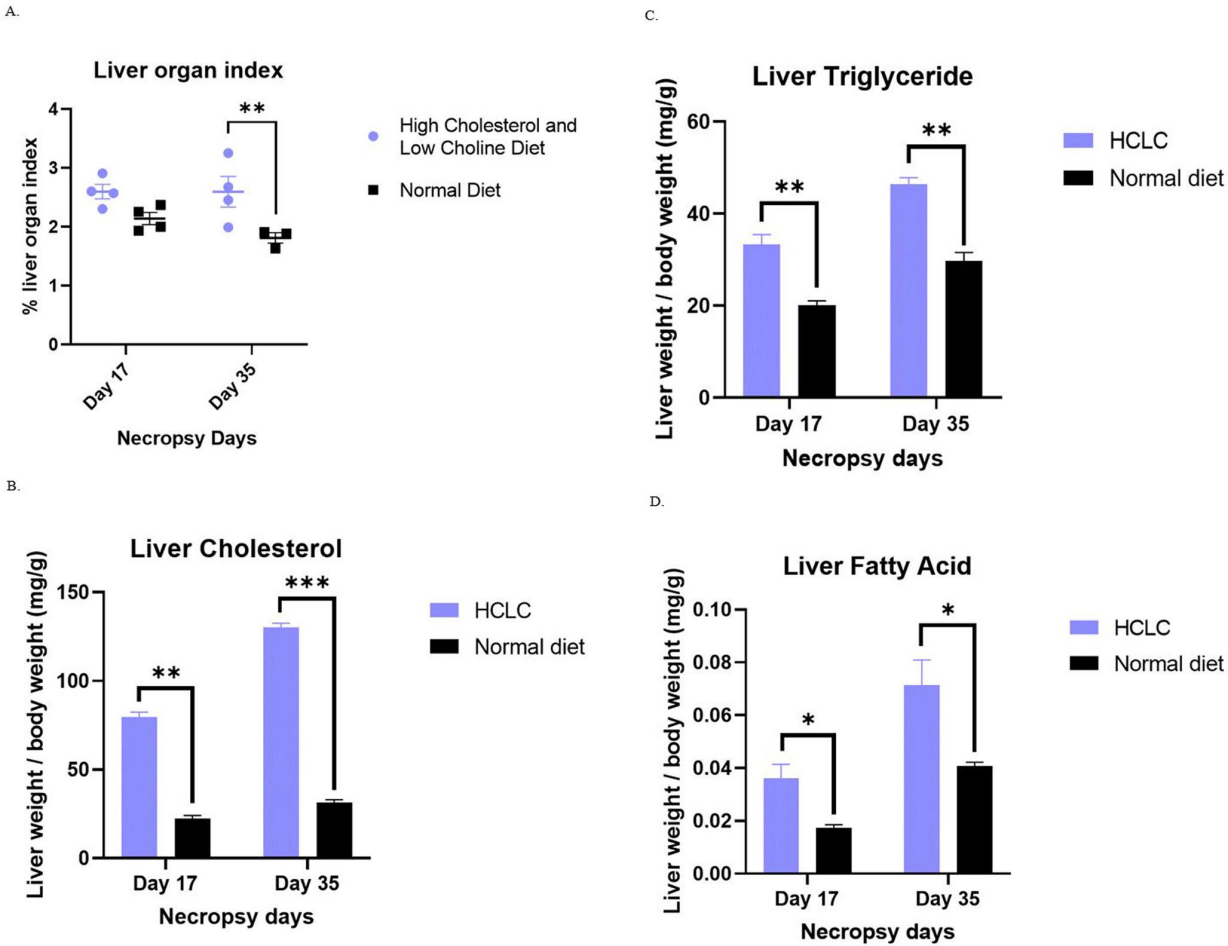
HCLC induced hepatic steatosis in white leghorn breed chickens. **A** An increase in the percentage of the liver organ index (LOI) in HCLC fed chickens was observed in comparison to regular diet fed chickens. The LOI is calculated by dividing the liver weight to total body weight and multiplying by 100. **B** The amount of cholesterol (CH), **C** triglycerides (TG) and **D** fatty acids in the liver of HCLC fed chicken was higher than that of normal diet fed chickens. The values for liver TG, CH and fatty acids were normalized with the liver weights. Data are shown as mean ± SD (*n* = 4, each necropsy time point). * *P* < 0.05, ** *P* < 0.01, *** *P* < 0.001; HCLC, high cholesterol and low choline diet; TG, triglycerides; CH, cholesterol

Birds were necropsied on day 17 and day 35 after changing to the experimental diet to evaluate liver specific lesions and lipid related measurements. White leghorn chickens fed HCLC diets demonstrated a significantly higher LOI on day 35 when compared to the normal diet fed chickens (2.6% vs 1.8%) (Fig. 2A). Although we found a higher LOI on day 17, the data was not significantly different between the groups (Fig. 2A). Liver cholesterol on day 17 (78 ± 4 vs 20 ± 3) and day 35 (130 ± 3 vs 28 ± 2) in the HCLC fed chickens was significantly higher than the normal diet fed chickens (Fig. 2B). Similarly, liver triglycerides on day 17 (33 ± 3 vs 20 ± 2) and day 35 (45 ± 3 vs 30 ± 3) (Fig. 2C) were significantly higher than the regular diet fed chickens. Liver fatty acids (Fig. 2D) were found to be significantly higher than the normal diet fed chickens on both day 17 (0.037 ± 0.004) and day 35 (0.077 ± 0.005).

### Excessive fat deposition and immune cells infiltration was seen in the livers of HCLC diet fed birds

**Fig. 3:**
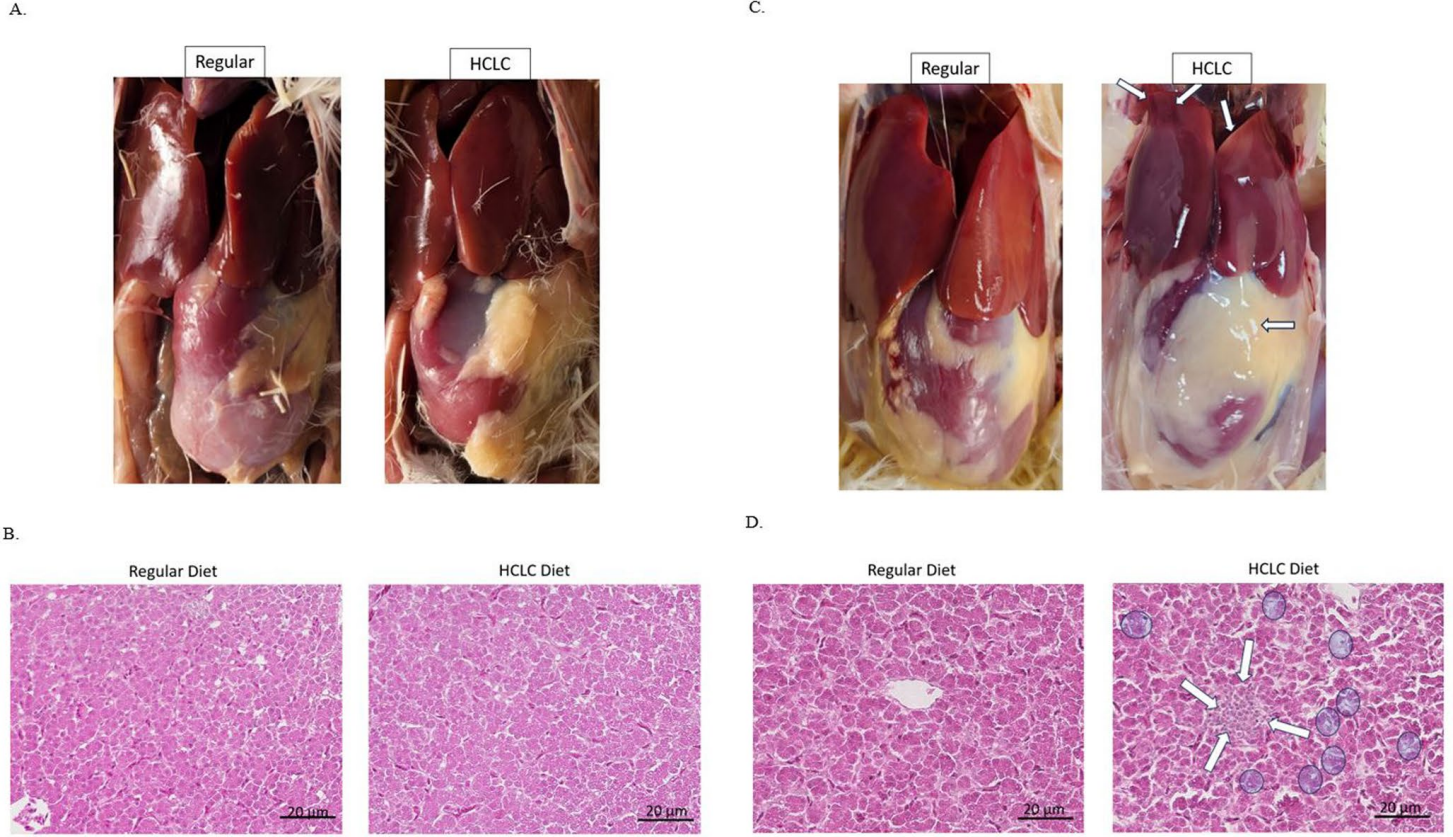
HCLC diet induced fat deposition and immune cells infiltration suggesting non-alcoholic hepatic steatosis. **A** Accumulation of abdominal fat in the coelomic cavity of chickens fed with HCLC diet at 17 days. No hemorrhages or paleness of the liver were recorded. **B** Liver of white leghorn chickens on day 17 fed with normal regular diet and/or HCLC diet were stained with hematoxylin & Eosin. No obvious differences are noted between the groups. Scale bar = 20 µm. **C** Pinpoint hepatic hemorrhages (arrows) and paleness with abundant intracelomic fat was observed in high cholesterol low choline diet (HCLC) fed birds in comparison to regular diet fed birds at 35 days. **D** Liver of white leghorn chickens on day 35 fed with regular diet and/or HCLC diet were stained with H & E. The white arrow indicates an area of immune cell infiltration. The circles denote the hepatic lipid vacuoles. HCLC, high cholesterol and low choline diet. Scale bar = 20 µm

The gross appearance of the liver did not change significantly between the groups by 17th day of the study. Although we did not observe intracelomic blood clots, which are commonly associated with FLHS, intracelomic fat accumulation was observed even at day 17 (Fig. 3A). There were no changes in the liver histologically between the groups on day 17 (Fig. 3B). Grossly, livers from HCLC fed birds were reported as large, friable, and varied in color from light yellow to orange around the borders of the liver on day 35. Pinpoint hemorrhages were visible on the liver capsule (Fig. 3C). Histological sections of liver demonstrated immune cells infiltration and ballooning on day 35 in the HCLC fed birds (Fig. 3D).

## Discussion

Our study describes the development of fatty liver disease in white leghorn chickens when a diet composed of higher concentration of cholesterol and lower concentration of choline is fed to chickens. Observed body weight gain, higher LOI, hyperlipidemia, hepatic steatosis, and immune cell infiltration after feeding chickens the HCLC diet demonstrates an easy and rapid diet-induced fatty liver disease model in chickens. Our study demonstrates similar findings as seen during NAFLD in humans characterized by weight gain, inflamed liver, hyperlipidemia, and nonalcoholic steatohepatitis (NASH) implying the model’s potential use to study human NAFLD in future studies.

The major organ for choline metabolism is the liver (Griffin et al. 1992). Phosphatidylcholine is made from choline in liver and is required for the assembly and secretion of very low-density lipoprotein (VLDL) (Cole et al. 2012). Thus, impairment in the synthesis and regulation of VLDL causes NAFLD (Masoodi et al. 2021). The development of NAFLD in rodents has been shown to be directly related to the level of cholesterol (Mueller et al. 2021; Parthasarathy et al. 2020). Our study demonstrates that an HCLC diet leads to high TG and cholesterol supporting the previous literature on the role of choline and cholesterol in the development of fatty liver. Thus, the observed effects in the white leghorn breed of chicken can be attributed to the combinatorial effect of HCLC. Previous studies identified that high-fat, high-fructose diets very closely recapitulate the human phenotype of NAFLD (Im et al. 2021). The HCLC diet used in our chicken model illustrates a similar phenotype as seen in humans.

ISA brown chickens and Plymouth rock chickens also demonstrated hyperlipidemia and hepatic steatosis when fed with low protein, high fat, high cholesterol, and low choline diets (LPHFCLC) (Lin et al. 2021). Consistent with our results, they also demonstrated the infiltration of immune cells in the liver. This suggests that diet-based fatty liver disease is not limited to one specific breed of chickens. In general, hens develop fatty liver disease naturally when they are actively laying (Shini et al. 2019) demonstrating an increased blood lipid level and lipid deposition in the liver (You et al. 2023). Similar to our findings in diet induced fatty liver disease, liver TG has been previously reported to be significantly higher in FLHS afflicted laying hens (Miao et al. 2021). Advanced approaches such as lipidomic analysis also reported an increase in the TG in the liver of FLHS hens (You et al. 2023). Thus, higher TG demonstrated in the liver and blood in our chicken model resembles FLHS in laying hens. Hence, our rapid chicken model can be used to test fatty liver disease specific therapeutics that could help the poultry industry develop long term solutions for FLHS.

Our HCLC chicken model can be used to study the emerging pathogen HEV. Mammalian animal models infected by HEV do not demonstrate liver specific lesions as seen in humans (Yadav and Kenney 2022, 2023). Chickens are the only animal model mimicking liver pathology as observed in humans when infected by HEV. The HCLC chicken model can be used to correlate NAFLD and HEV comorbidity mechanisms. Currently, there are multiple animal models that are used for examining NAFLD in humans including mice, rats, rabbits, ossabaw pigs, macaques, and zebrafish (Soret et al. 2020). However, there are several disadvantages to using these animal models. Some of the animals cannot become obese, which is the most common risk factor for NAFLD patients. In our HCLC chicken model, we demonstrate significant weight gain in the HCLC fed birds in comparison to normal diet fed birds. Some animal models require large time investments and cost, a high price for chemicals, a need for highly trained personnel, and advanced genetic manipulations. Our study describes a cost-effective, time-efficient, and easier to use fatty liver disease chicken model that could help to study hepatitis-causing viral diseases occurring during fatty liver disease comorbidity.

Many discoveries relevant to human health have been made using chicken models. Chickens were used as a model to study human ovarian cancer (Johnson and Giles 2013). A chicken model was initially used in the field of embryology and developmental biology (Stern 2005). Chicken models were very useful in studying the underlying mechanisms involved in Zika virus infections (Ambagala et al. 2020). Discovery of B lymphocytes was initially achieved by studying the bursa of fabricus in chicken (Taylor and McCorkle 2009) that led to the concept of B and T cells forming the basis of adaptive immunity in humans. For decades, chickens have been used as a model to study obesity, adipose biology, and insulin resistance (Mellouk et al. 2018). Our findings demonstrate similar metabolic syndromes as seen in humans during NAFLD such as obesity, hyperlipidemia, hypercholesterolemia, and hypertriglyceridemia. Similar results were described in the hens that were fed a high-energy and low choline diet (Lv et al. 2018). However, the study used 80-week-old JINGFEN 1 laying hen compared to 3-week-old white leghorn chicken irrespective of sex used in our study. Another study described the use of similar high energy low protein diet in the 63-week-old Hyline Brown laying hens to produce diet induced fatty liver disorder demonstrating lipid metabolism and skeletal health axis (Jiang et al. 2013). Hence, chickens appear to be an interesting model to elucidate critical information on roles of lipids in hepatic and extrahepatic disorders when coinfected with liver tropic viruses. Future studies will be directed to understand the disorders in lipid metabolism when infected by HEV and its association with extrahepatic disorders by various factors such as viral and/or host immune responses (Zhang et al. 2019).

In conclusion, this HCLC diet-induced avian fatty liver disease model can be rapidly generated for basic and pre-clinical research. In addition, multiple metabolic disorders such as diabetes, cardiovascular disease, and several pathogens induced hepatitis caused by hepatitis E, *Salmonella* species, *E. coli,* and *Campylobacter* species, which are correlated with NAFLD, can be studied utilizing this fatty liver disease model. Thus, our chicken model would be beneficial to screen different treatment regimens against NAFLD in humans and fatty liver syndrome in chickens.

### Electronic supplementary material

Below is the link to the electronic supplementary material.

Supplementary Material 1

## Data Availability

All the data pertaining to the study is presented in the manuscript.

## References

[CR1] Ambagala A, Truong T, Cottam-Birt C, Berhane Y, Gerdts V, Karniychuk U, ... Babiuk S (2020) Susceptibility of chicken embryos, sheep, cattle, pigs, and chickens to zika virus infection. Front Vet Sci 7:23. 10.3389/fvets.2020.00023 10.3389/fvets.2020.00023 PMC7012786 32118055

[CR2] Ayala I, Castillo AM, Adánez G, Fernández-Rufete A, Pérez BG, Castells MT (2009) Hyperlipidemic chicken as a model of non-alcoholic steatohepatitis. Exp Biol Med (Maywood) 234(1):10–16. 10.3181/0807-rm-219 10.3181/0807-RM-219 18997102

[CR3] Baumeier C, Schlüter L, Saussenthaler S, Laeger T, Rödiger M, Alaze SA, ... Schürmann A (2017) Elevated hepatic DPP4 activity promotes insulin resistance and non-alcoholic fatty liver disease. Mol Metab 6(10):1254–1263. 10.1016/j.molmet.2017.07.016 10.1016/j.molmet.2017.07.016 PMC5641684 29031724

[CR4] Bergen WG, Mersmann HJ (2005) Comparative aspects of lipid metabolism: impact on contemporary research and use of animal Models1. J Nutr 135(11):2499–2502. 10.1093/jn/135.11.2499 16251600 10.1093/jn/135.11.2499

[CR5] Caldwell SH, Lee VD, Kleiner DE, Al-Osaimi AM, Argo CK, Northup PG, Berg CL (2009) NASH and cryptogenic cirrhosis: a histological analysis. Ann Hepatol 8(4):346–352 20009134 10.1016/S1665-2681(19)31748-X PMC8381243

[CR6] Chalasani N, Younossi Z, Lavine JE, Charlton M, Cusi K, Rinella M, ... Sanyal AJ (2018) The diagnosis and management of nonalcoholic fatty liver disease: practice guidance from the American Association for the Study of Liver Diseases. Hepatology 67(1):328–357 10.1002/hep.29367 28714183

[CR7] Cole LK, Vance JE, Vance DE (2012) Phosphatidylcholine biosynthesis and lipoprotein metabolism. Biochim Biophys Acta 1821(5):754–761. 10.1016/j.bbalip.2011.09.009 21979151 10.1016/j.bbalip.2011.09.009

[CR8] Corbin KD, Zeisel SH (2012) Choline metabolism provides novel insights into nonalcoholic fatty liver disease and its progression. Curr Opin Gastroenterol 28(2):159–165. 10.1097/MOG.0b013e32834e7b4b 22134222 10.1097/MOG.0b013e32834e7b4b PMC3601486

[CR9] Cotter TG, Rinella M (2020) Nonalcoholic fatty liver disease 2020: the state of the disease. Gastroenterology 158(7):1851–1864. 10.1053/j.gastro.2020.01.052 32061595 10.1053/j.gastro.2020.01.052

[CR10] Craig M, Yarrarapu SNS, Dimri M, Biochemistry, Cholesterol (2023) In: StatPearls [Internet]. Treasure Island (FL): StatPearls Publishing; 2024 Jan. Available from: https://www.ncbi.nlm.nih.gov/books/NBK513326/30020698

[CR11] Dai XY, Zhu SY, Chen J, Li MZ, Zhao Y, Talukder M, Li JL (2022) Lycopene alleviates di(2-ethylhexyl) phthalate-induced splenic injury by activating P62-Keap1-NRF2 signaling. Food Chem Toxicol 168:113324. 10.1016/j.fct.2022.113324 35917956 10.1016/j.fct.2022.113324

[CR12] Davern TJ, Chalasani N, Fontana RJ, Hayashi PH, Protiva P, Kleiner DE,... Hoofnagle JH (2011) Acute hepatitis E infection accounts for some cases of suspected drug-induced liver injury. Gastroenterology 141(5):1665–1672.e1661–1669. 10.1053/j.gastro.2011.07.051 10.1053/j.gastro.2011.07.051 PMC3654540 21855518

[CR13] Du T, Sun X, Yuan G, Zhou X, Lu H, Lin X, Yu X (2016) Lipid phenotypes in patients with nonalcoholic fatty liver disease. Metabolism 65(9):1391–1398 27506745 10.1016/j.metabol.2016.06.006

[CR14] Estes C, Razavi H, Loomba R, Younossi Z, Sanyal AJ (2018) Modeling the epidemic of nonalcoholic fatty liver disease demonstrates an exponential increase in burden of disease. Hepatology 67(1):123–133 28802062 10.1002/hep.29466 PMC5767767

[CR15] Flessa CM, Nasiri-Ansari N, Kyrou I, Leca BM, Lianou M, Chatzigeorgiou A,... Randeva HS (2022) Genetic and diet-induced animal models for non-alcoholic fatty liver disease (NAFLD) research. Int J Mol Sci 23(24). 10.3390/ijms232415791 10.3390/ijms232415791 PMC9780957 36555433

[CR16] Gao X, Liu P, Wu C, Wang T, Liu G, Cao H,... Guo X (2019) Effects of fatty liver hemorrhagic syndrome on the AMP-activated protein kinase signaling pathway in laying hens. Poult Sci 98(5):2201–2210. 10.3382/ps/pey586 10.3382/ps/pey586 30608557

[CR17] Griffin HD, Guo K, Windsor D, Butterwith SC (1992) Adipose tissue lipogenesis and fat deposition in leaner broiler chickens. J Nutr 122(2):363–368. 10.1093/jn/122.2.363 1732477 10.1093/jn/122.2.363

[CR18] Im YR, Hunter H, de Gracia Hahn D, Duret A, Cheah Q, Dong J,... Mann JP (2021) A systematic review of animal models of NAFLD Finds high-fat, high-fructose diets most closely resemble human NAFLD. Hepatology 74(4):1884–1901. 10.1002/hep.31897 10.1002/hep.31897 33973269

[CR19] Jiang L, Yang F, Liao H, Chen W, Dai X, Peng C,... Cao H (2024) Molybdenum and cadmium cause blood-testis barrier dysfunction through ROS-mediated NLRP3 inflammasome activation in sheep. Sci Total Environ 906:167267. 10.1016/j.scitotenv.2023.167267 10.1016/j.scitotenv.2023.167267 37741404

[CR20] Jiang S, Cheng HW, Cui LY, Zhou ZL, Hou JF (2013) Changes of blood parameters associated with bone remodeling following experimentally induced fatty liver disorder in laying hens. Poult Sci 92(6):1443–1453. 10.3382/ps.2012-02800 23687138 10.3382/ps.2012-02800

[CR21] Johnson PA, Giles JR (2013) The hen as a model of ovarian cancer. Nat Rev Cancer 13(6):432–436. 10.1038/nrc3535 23676850 10.1038/nrc3535

[CR22] Kenney SP, Meng XJ (2019) Hepatitis E virus: animal models and zoonosis. Annu Rev Anim Biosci 7:427–448. 10.1146/annurev-animal-020518-115117 30285462 10.1146/annurev-animal-020518-115117

[CR23] Kim WR, Lake JR, Smith JM, Schladt DP, Skeans MA, Harper AM,... Kasiske BL (2018) OPTN/SRTR 2016 annual data report: liver. Am J Transplant 18(S1):172–253. 10.1111/ajt.14559 10.1111/ajt.14559 29292603

[CR24] Laliotis GP, Bizelis I, Rogdakis E (2010) Comparative approach of the de novo Fatty Acid Synthesis (Lipogenesis) between ruminant and non ruminant mammalian species: from biochemical level to the main regulatory lipogenic genes. Curr Genomics 11(3):168–183. 10.2174/138920210791110960 21037855 10.2174/138920210791110960 PMC2878982

[CR25] Lenggenhager D, Pawel S, Honcharova-Biletska H, Evert K, Wenzel JJ, Montani M,... Weber A (2021) The histologic presentation of hepatitis E reflects patients' immune status and pre-existing liver condition. Mod Pathol 34(1):233–248. 10.1038/s41379-020-0593-1 10.1038/s41379-020-0593-1 PMC7806507 32572157

[CR26] Letexier D, Pinteur C, Large V, Fréring V, Beylot M (2003) Comparison of the expression and activity of the lipogenic pathway in human and rat adipose tissue. J Lipid Res 44(11):2127–2134. 10.1194/jlr.M300235-JLR200 12897191 10.1194/jlr.M300235-JLR200

[CR27] Li TC, Wakita T (2019) Small animal models of Hepatitis E virus infection. Cold Spring Harb Perspect Med 9(8). 10.1101/cshperspect.a032581 10.1101/cshperspect.a032581 PMC6671932 29735581

[CR28] Lin CW, Huang TW, Peng YJ, Lin YY, Mersmann HJ, Ding ST (2021) A novel chicken model of fatty liver disease induced by high cholesterol and low choline diets. Poult Sci 100(3):100869. 10.1016/j.psj.2020.11.046 33516481 10.1016/j.psj.2020.11.046 PMC7936157

[CR29] Lv Z, Xing K, Li G, Liu D, Guo Y (2018) Dietary genistein alleviates lipid metabolism disorder and inflammatory response in laying hens with fatty liver syndrome. Front Physiol 9:1493. 10.3389/fphys.2018.01493 30405443 10.3389/fphys.2018.01493 PMC6207982

[CR30] Masoodi M, Gastaldelli A, Hyötyläinen T, Arretxe E, Alonso C, Gaggini M, ... Orešič M (2021) Metabolomics and lipidomics in NAFLD: biomarkers and non-invasive diagnostic tests. Nat Rev Gastroenterol Hepatol 18(12):835–856. 10.1038/s41575-021-00502-9 10.1038/s41575-021-00502-9 34508238

[CR31] Matsuzawa N, Takamura T, Kurita S, Misu H, Ota T, Ando H, ... Kaneko S (2007) Lipid-induced oxidative stress causes steatohepatitis in mice fed an atherogenic diet. Hepatology 46(5):1392–1403. 10.1002/hep.21874 10.1002/hep.21874 17929294

[CR32] Mellouk N, Ramé C, Barbe A, Grandhaye J, Froment P, Dupont J (2018) Chicken is a useful model to investigate the role of adipokines in metabolic and reproductive diseases. Int J Endocrinol 2018:4579734. 10.1155/2018/4579734 30018639 10.1155/2018/4579734 PMC6029501

[CR33] Miao YF, Gao XN, Xu DN, Li MC, Gao ZS, Tang ZH, ... Song SQ (2021) Protective effect of the new prepared Atractylodes macrocephala Koidz polysaccharide on fatty liver hemorrhagic syndrome in laying hens. Poult Sci 100(2):938–948. 10.1016/j.psj.2020.11.036 10.1016/j.psj.2020.11.036 PMC7858188 33518147

[CR34] Mueller AM, Kleemann R, Gart E, van Duyvenvoorde W, Verschuren L, Caspers M, ... Morrison MC (2021) Cholesterol accumulation as a driver of hepatic inflammation under translational dietary conditions can be attenuated by a multicomponent medicine. Front Endocrinol (Lausanne) 12:601160. 10.3389/fendo.2021.601160 10.3389/fendo.2021.601160 PMC8014004 33815271

[CR35] Parthasarathy G, Revelo X, Malhi H (2020) Pathogenesis of nonalcoholic steatohepatitis: an overview. Hepatol Commun 4(4):478–492. 10.1002/hep4.1479 32258944 10.1002/hep4.1479 PMC7109346

[CR36] Ramalho de Lima M, Kaneko IN, de Lima AV, de Melo LN, de Lima MC, de Brito A, ... Marimuthu S (2024) Choline supplementation: impact on broiler chicken performance, steatosis, and economic viability from from 1 to 42 days. PLoS ONE 19(3):e0295488. 10.1371/journal.pone.0295488 10.1371/journal.pone.0295488 PMC10950236 38502648

[CR37] Ravindran V, Tancharoenrat P, Zaefarian F, Ravindran G (2016) Fats in poultry nutrition: digestive physiology and factors influencing their utilisation. Anim Feed Sci Technol 213:1–21. 10.1016/j.anifeedsci.2016.01.012 10.1016/j.anifeedsci.2016.01.012

[CR38] Rozenboim I, Mahato J, Cohen NA, Tirosh O (2016) Low protein and high-energy diet: a possible natural cause of fatty liver hemorrhagic syndrome in caged White Leghorn laying hens. Poult Sci 95(3):612–621. 10.3382/ps/pev367 26755655 10.3382/ps/pev367

[CR39] Savard C, Tartaglione EV, Kuver R, Haigh WG, Farrell GC, Subramanian S, ... Ioannou GN (2013) Synergistic interaction of dietary cholesterol and dietary fat in inducing experimental steatohepatitis. Hepatology 57(1):81–92. 10.1002/hep.25789 10.1002/hep.25789 PMC5341743 22508243

[CR40] Shini A, Shini S, Bryden W (2019) Fatty liver haemorrhagic syndrome occurrence in laying hens: impact of production system. Avian Pathol 48(1):25–34 30345810 10.1080/03079457.2018.1538550

[CR41] Soret PA, Magusto J, Housset C, Gautheron J (2020) In vitro and in vivo models of non-alcoholic fatty liver disease: a critical appraisal. J Clin Med 10(1). 10.3390/jcm10010036 10.3390/jcm10010036 PMC7794936 33374435

[CR42] Stern CD (2005) The chick; a great model system becomes even greater. Dev Cell 8(1):9–17. 10.1016/j.devcel.2004.11.018 15621526 10.1016/j.devcel.2004.11.018

[CR43] Subramanian S, Goodspeed L, Wang S, Kim J, Zeng L, Ioannou GN, ... Chait A (2011) Dietary cholesterol exacerbates hepatic steatosis and inflammation in obese LDL receptor-deficient mice. J Lipid Res 52(9):1626–1635. 10.1194/jlr.M016246 10.1194/jlr.M016246 PMC3151683 21690266

[CR44] Taylor RL Jr, McCorkle FM Jr (2009) A landmark contribution to poultry science–immunological function of the bursa of Fabricius. Poult Sci 88(4):816–823. 10.3382/ps.2008-00528 19276427 10.3382/ps.2008-00528

[CR45] Trott KA, Giannitti F, Rimoldi G, Hill A, Woods L, Barr B, ... Mete A (2014) Fatty liver hemorrhagic syndrome in the backyard chicken: a retrospective histopathologic case series. Vet Pathol 51(4):787–795. 10.1177/0300985813503569 10.1177/0300985813503569 24091813

[CR46] Tsai MT, Chen YJ, Chen CY, Tsai MH, Han CL, Chen YJ, ... Ding ST (2017) Identification of potential plasma biomarkers for nonalcoholic fatty liver disease by integrating transcriptomics and proteomics in laying hens. J Nutr 147(3):293–303. 10.3945/jn.116.240358 10.3945/jn.116.240358 28077733

[CR47] Yadav KK, Kenney SP (2022) Hepatitis E virus zoonotic axis. In: Sing A (ed) Zoonoses: infections affecting humans and animals, pp 1-28. Springer International Publishing, Cham

[CR48] Yadav KK, Kenney SP (2023) Animal Models for studying congenital transmission of Hepatitis E virus. Microorganisms 11(3). 10.3390/microorganisms11030618 10.3390/microorganisms11030618 PMC10057890 36985191

[CR49] You M, Zhang S, Shen Y, Zhao X, Chen L, Liu J, Ma N (2023) Quantitative lipidomics reveals lipid perturbation in the liver of fatty liver hemorrhagic syndrome in laying hens. Poult Sci 102(2):102352. 10.1016/j.psj.2022.102352 36473380 10.1016/j.psj.2022.102352 PMC9723938

[CR50] Younossi ZM, Koenig AB, Abdelatif D, Fazel Y, Henry L, Wymer M (2016) Global epidemiology of nonalcoholic fatty liver disease—meta-analytic assessment of prevalence, incidence, and outcomes. Hepatology 64(1):73–84 26707365 10.1002/hep.28431

[CR51] Younossi ZM, Stepanova M, Younossi Y, Golabi P, Mishra A, Rafiq N, Henry L (2020) Epidemiology of chronic liver diseases in the USA in the past three decades. Gut 69(3):564–568 31366455 10.1136/gutjnl-2019-318813

[CR52] Yu Z, Cheng M, Luo S, Wei J, Song T, Gong Y, Zhou Z (2023) Comparative lipidomics and metabolomics reveal the underlying mechanisms of taurine in the alleviation of nonalcoholic fatty liver disease using the aged laying hen model. Mol Nutr Food Res 67(24):e2200525. 10.1002/mnfr.202200525 37909476 10.1002/mnfr.202200525

[CR53] Zhang JW, Chen DW, Yu B, Wang YM (2011) Effect of dietary energy source on deposition and fatty acid synthesis in the liver of the laying hen. Br Poult Sci 52(6):704–710. 10.1080/00071668.2010.547457 22221236 10.1080/00071668.2010.547457

[CR54] Zhang Y, Li G, Zhao Y, Dai X, Hu M, Cao H,... Yang F (2023) Inhibition of calcium imbalance protects hepatocytes from vanadium exposure-induced inflammation by mediating mitochondrial-associated endoplasmic reticulum membranes in ducks. Poult Sci 102(12):103013. 10.1016/j.psj.2023.103013 10.1016/j.psj.2023.103013 PMC10591013 37856907

[CR55] Zhang YZ, Zhang ZM, Zhou LT, Zhu J, Zhang XH, Qi W,... Ye L (2019) Di (2-ethylhexyl) phthalate disorders lipid metabolism via TYK2/STAT1 and autophagy in rats. Biomed Environ Sci 2(6): 406–418. 10.3967/bes2019.055 10.3967/bes2019.055 31262386

[CR56] Zheng S, Hoos L, Cook J, Tetzloff G, Davis H Jr, van Heek M, Hwa JJ (2008) Ezetimibe improves high fat and cholesterol diet-induced non-alcoholic fatty liver disease in mice. Eur J Pharmacol 584(1):118–124. 10.1016/j.ejphar.2008.01.045 18329014 10.1016/j.ejphar.2008.01.045

