# Supplementary material for: Development of fatty liver disease model using high cholesterol and low choline diet in white leghorn chickens
Source: Vet Res Commun. 2024 Jun 11;48(4):2489–97. doi: 10.1007/s11259-024-10420-1 (PMC11315703; doi:10.1007/s11259-024-10420-1)
Supplement: Supplementary file 1 — Supplementary Material 1 [file 11259_2024_10420_MOESM1_ESM.docx]

**Supplement to:** Development of avian fatty liver disease model using white leghorn chickens.

**S1 - High cholesterol and low choline diet**

**Table S1.1. High cholesterol and low choline diet (ingredient %)**

|  | Cornmeal | 65.761 |
| --- | --- | --- |
|  | Soybean protein | 7.892 |
|  | Soybean meal | 9.647 |
|  | Wheat bran | 4.382 |
|  | Rice bran | 4.382 |
|  | CaCO_3_ | 2.196 |
|  | CaHPO_4_ | 1.755 |
|  | Beef tallow | 1.314 |
|  | NaCl | 0.353 |
|  | DL^-^ Methionine | 0.353 |
|  | Premix | 0.005 |
|  | Choline | - |
|  | Cholesterol | 1.961 |
|  | Total | 100 |

**Table S1.2. Premix supplied per kg of diet:**

|  | Vit A | 1.8 mg |
| --- | --- | --- |
|  | Vit D_3_ | 0.005 mg |
|  | Vit E | 9.09 mg |
|  | Vit K | 0.5 mg |
|  | Vit B_12_ | 0.007 ug |
|  | Pantothenic acid | 2.99 mg |
|  | Riboflavin | 1.63 mg |
|  | Cu | 1.25 mg |
|  | Mn | 24.06 mg |
|  | Zn | 12.7 mg |
|  | Se | 0.06 mg |
|  | Iodide | 0.35 mg |

**Table S1.3. Nutrient composition (%)**

|  | Crude protein | 16.94 |
| --- | --- | --- |
|  | Fat | 7.55 |
|  | Moisture | 10.54 |
|  | Ash | 5.14 |
|  | Methionine | 0.63 |
|  | Lysine | 0.94 |
|  | Choline | 0.08 |
|  | Cholesterol | 2 |
|  | Calculated metabolic energy (kcal/kg) | 3,043.20 |

**S2 - Control diet**

**Table S2.1. Control diet (ingredient %)**

| 1. | Cornmeal | 67.043 |
| --- | --- | --- |
| 2. | Soybean protein | 8.046 |
| 3. | Soybean meal | 9.835 |
| 4. | Wheat bran | 4.468 |
| 5. | Rice bran | 4.468 |
| 6. | CaCO_3_ | 2.239 |
| 7. | CaHPO_4_ | 1.789 |
| 8. | Beef tallow | 1.339 |
| 9. | NaCl | 0.360 |
| 10. | DL^-^ Methionine | 0.360 |
| 11. | Premix | 0.005 |
| 12. | Choline | 0.05 |
| 13. | Cholesterol | - |
|  | Total | 100 |

**Table S2.2. Premix supplied per kg of diet:**

| 1. | Vit A | - 1. mg |
| --- | --- | --- |
| 2. | Vit D_3_ |  |
| 3. | Vit E | - 1. g |
| 4. | Vit K | - 1. mg |
| 5. | Vit B_12_ | 0.007 ug |
| 6. | Pantothenic acid | - 1. g |
| 7. | Riboflavin | - 1. g |
| 8. | Cu | - 1. g |
| 9. | Mn | - 1. g |
| 10. | Zn | - 1. mg |
| 11. | Se | - 1. g |
| 12. | Iodide | 0.35 mg |

**Table S2.3. Nutrient composition (%)**

| 1. | Crude protein | 16.55 |
| --- | --- | --- |
| 2. | Fat | 5.28 |
| 3. | Moisture | 11.48 |
| 4. | Ash | 5.39 |
| 5. | Methionine | 0.63 |
| 6. | Lysine | 0.94 |
| 7. | Choline | 0.13 |
| 8. | Cholesterol | 0 |
|  | Calculated metabolic energy (kcal/kg) | 3,043.20 |
